# Serum cortisol is negatively related to hippocampal volume, brain structure, and memory performance in healthy aging and Alzheimer’s disease

**DOI:** 10.3389/fnagi.2023.1154112

**Published:** 2023-05-12

**Authors:** Julian Dronse, Anna Ohndorf, Nils Richter, Gérard N. Bischof, Ronja Fassbender, Qumars Behfar, Hannes Gramespacher, Kim Dillen, Heidi I. L. Jacobs, Juraj Kukolja, Gereon R. Fink, Oezguer A. Onur

**Affiliations:** ^1^Cognitive Neuroscience, Institute of Neuroscience and Medicine (INM-3), Jülich Research Centre, Jülich, Germany; ^2^Department of Neurology, Faculty of Medicine and University Hospital Cologne, University of Cologne, Cologne, Germany; ^3^Department of Nuclear Medicine, Multimodal Neuroimaging Group, Faculty of Medicine and University Hospital Cologne, University of Cologne, Cologne, Germany; ^4^Department of Palliative Medicine, Multimodal Neuroimaging Group, Faculty of Medicine and University Hospital Cologne, University of Cologne, Cologne, Germany; ^5^Department of Radiology, Gordon Center for Medical Imaging, Massachusetts General Hospital, Harvard Medical School, Boston, MA, United States; ^6^School for Mental Health and Neuroscience, Alzheimer Centre Limburg, Maastricht University, Maastricht, Netherlands; ^7^Department of Neurology and Clinical Neurophysiology, Helios University Hospital Wuppertal, Wuppertal, Germany; ^8^Faculty of Health Witten/Herdecke University, Witten, Germany

**Keywords:** dementia, glucocorticoids, mild cognitive impairment (MCI), atrophy, voxel-based morphometry (VBM), Hippocampus, memory, cortisol

## Abstract

**Objective:**

Elevated cortisol levels have been frequently reported in Alzheimer’s disease (AD) and linked to brain atrophy, especially of the hippocampus. Besides, high cortisol levels have been shown to impair memory performance and increase the risk of developing AD in healthy individuals. We investigated the associations between serum cortisol levels, hippocampal volume, gray matter volume and memory performance in healthy aging and AD.

**Methods:**

In our cross-sectional study, we analyzed the relationships between morning serum cortisol levels, verbal memory performance, hippocampal volume, and whole-brain voxel-wise gray matter volume in an independent sample of 29 healthy seniors (HS) and 29 patients along the spectrum of biomarker-based AD.

**Results:**

Cortisol levels were significantly elevated in patients with AD as compared to HS, and higher cortisol levels were correlated with worse memory performance in AD. Furthermore, higher cortisol levels were significantly associated with smaller left hippocampal volumes in HS and indirectly negatively correlated to memory function through hippocampal volume. Higher cortisol levels were further related to lower gray matter volume in the hippocampus and temporal and parietal areas in the left hemisphere in both groups. The strength of this association was similar in HS and AD.

**Conclusion:**

In AD, cortisol levels are elevated and associated with worse memory performance. Furthermore, in healthy seniors, higher cortisol levels show a detrimental relationship with brain regions typically affected by AD. Thus, increased cortisol levels seem to be indirectly linked to worse memory function even in otherwise healthy individuals. Cortisol may therefore not only serve as a biomarker of increased risk for AD, but maybe even more importantly, as an early target for preventive and therapeutic interventions.

## 1. Introduction

With the repeated rather disappointing outcome of symptomatic and causal treatments, a large body of Alzheimer’s disease (AD) research has focused on early prediction, preventive treatments, and modifiable lifestyle factors. One of these factors is stress [for recent reviews see (Justice, 2018; Canet et al., 2019; Ouanes and Popp, 2019)]. One of the many neurobiological correlates of stress is the excretion of cortisol from the adrenal glands, regulated *via* a complex cascade of feedback mechanisms termed the hypothalamus-pituitary–adrenal (HPA) axis (Smith and Vale, 2006). Damage to hippocampal neurons after elevated glucocorticoid exposure was first reported in guinea pigs (der Mühlen and Ockenfels, 1968) and has since been replicated in other rodents (Sapolsky, 1985) and primates (Sapolsky et al., 1990). Notably, the hippocampus contains the highest number of glucocorticoid receptors in the rodent brain (McEwen et al., 1968) and is involved in a negative feedback loop to the HPA axis (Jacobson and Sapolsky, 1991). The latter findings led to the so-called ‘glucocorticoid cascade hypothesis’ (Sapolsky et al., 1986), which posits that elevated levels of glucocorticoids damage the hippocampus, resulting in a lack of inhibitory control over the HPA axis, consecutively leading to even higher glucocorticoid levels and further hippocampal damage. Consistent with this hypothesis, more than 30 years ago, cortisol levels in AD were found to be elevated and linked to hippocampal atrophy, reductions in cerebral metabolism, and dementia severity (Davis et al., 1986; de Leon et al., 1988). Since then, elevated cortisol levels in patients with AD have been confirmed in various biofluids, including cerebrospinal fluid (CSF) *in vivo* (Popp et al., 2009, 2015) and postmortem (Swaab et al., 1994; Hoogendijk et al., 2006), plasma (Umegaki et al., 2000), serum (Lara et al., 2013), urine (Ennis et al., 2017), and saliva (Giubilei et al., 2001).

As a possible factor in AD etiology, cortisol levels have been linked to amyloid-β (Aβ) – the main pathological hallmark of AD – as assessed by [^11^C]Pittsburgh Compound-B (PIB)-PET (Toledo et al., 2012) and serum Aβ 1–42 (Ishijima et al., 2018), and have also been shown to mediate adverse effects of amyloid-β on cognition in healthy older subjects (Pietrzak et al., 2017). Animal models have demonstrated that glucocorticoids increase Aβ and tau accumulation (Green et al., 2006) and the proportion of the more toxic Aβ 1–42 relative to Aβ 1–40 (Kulstad et al., 2005). Also, Aβ injection leads to progressive HPA-axis-dysregulation in rats (Brureau et al., 2013). Ultimately, high cortisol levels have been linked to smaller global and regional gray matter volumes and impaired cognitive functioning (Geerlings et al., 2015; Echouffo-Tcheugui et al., 2018), cognitive decline (Lupien et al., 1994), and progressive hippocampal atrophy (Lupien et al., 1998), as well as to the future onset of AD in otherwise healthy individuals (Hinterberger et al., 2013; Ennis et al., 2017), and the clinical progression of established AD (Csernansky et al., 2006; Huang et al., 2009; Popp et al., 2015).

While some data indicate that these associations are specific to the hippocampus – i.e., not significantly affecting whole brain atrophy (Lawlor et al., 1994), frontal atrophy (Huang et al., 2009), or other temporal areas (Lupien et al., 1998) – there also have been contradictory results with no significant hippocampal involvement (Kremen et al., 2010; Echouffo-Tcheugui et al., 2018). Also, null-findings have been reported showing no relationship between cortisol, cognitive functioning, and dementia (Schrijvers et al., 2011; Singh-Manoux et al., 2014).

Reasons for these discrepancies may be that most previous studies relied on clinical criteria and did not use biomarker-based AD diagnosis – possibly including AD mimics – with some only describing the broad syndrome of etiologically unspecified dementia. Further, while most studies examining stress-related atrophy or hippocampal volume used CT-based imaging and visual inspection, to the best of our knowledge, only three other recent studies have used voxel-wise MRI analysis to allow for a more objective and detailed assessment (Toledo et al., 2013; Echouffo-Tcheugui et al., 2018; Wirth et al., 2019) and none have jointly analyzed ROI volumes, voxel-wise MRI data and memory performance.

Accordingly, we aimed to determine the associations of serum cortisol levels with gray matter volume and memory performance in a sample of healthy older subjects and a patient group representing the entire AD spectrum as confirmed by CSF and PET biomarkers. To allow for a comprehensive assessment of cortisol effects on gray matter structure, we used voxel-wise whole-brain structural MRI analyses, as well as automated region of interest (ROI)-based techniques focusing on the hippocampus as a critical ROI.

## 2. Methods and materials

### 2.1. Participants

The sample used for the current analysis was part of a larger study in which a total of 171 healthy young subjects, healthy seniors (HS), subjects with subjective cognitive decline, and patients with prodromal AD (i.e., mild cognitive impairment due to AD) and AD dementia underwent a multimodal imaging protocol, a comprehensive neuropsychological assessment, and blood sampling. We selected healthy seniors and patients with AD with serum cortisol values and structural MRI available for the current analysis.

Healthy seniors were recruited from the general population and the local research facility (Jülich Research Centre) *via* online study advertisements. Patients were recruited from the memory clinic of the University Hospital of Cologne and data collection was part of their diagnostic workup. Participants were recruited between 50 and 80 years of age (current sample: 50–73 years, mean 64.6 years, SD 6.2 years), 39.7% of subjects were female (31% of healthy seniors and 48.3% of patients with AD). Healthy seniors had no deficits on neuropsychological testing according to normative data and no signs of depression according to the Hamilton Depression Rating Scale (HAM-D) (Hamilton, 1960). Prodromal AD and AD dementia were defined by the presence of objective memory impairment [using the delayed recall run of the Verbal Learning and Memory Test (Helmstaedter and Durwen, 1990)] at least 1.5 standard deviations below the mean of normative scores of a healthy older sample. Prodromal patients with AD had a mini-mental state examination (MMSE) score > 23 (Folstein et al., 1975) and intact activities of daily living as verified by an informant. The MMSE of AD dementia patients ranged between 15 and 22, and activities of daily living were compromised. Based on the recent notion that “prodromal AD” and “AD dementia” do not represent distinct entities, but two stages on a continuum of AD (Jack et al., 2018), both patient groups were combined for all analyses and are from here on referred to as “patients” or “AD group.”

The diagnosis was made according to standard diagnostic criteria (Albert et al., 2011; Dubois et al., 2014) and after interdisciplinary discussion (neurologists specialized in dementia care, clinical neuropsychologists, neuroradiologists, and nuclear medicine specialists). All patients had a biomarker profile indicative of AD (assessed by CSF analysis or PET), which was part of their clinical workup. CSF biomarker positivity was determined using a cut-off on the tau/Aβ 1–42 ratio of >0.52 (Duits et al., 2014). Thirteen patients received [^11^C]PIB or [^18^F]Florbetapir amyloid PET and [^18^F]AV-1451 tau PET additionally to (12 patients) or instead of (1 patient) CSF analysis. Patients had a pattern of amyloid and tau deposition typical of AD as determined by a nuclear medicine specialist. Two patients had a CSF tau/Aβ 1–42 ratio < 0.52 but typical AD patterns on tau and amyloid PET and were, therefore, included in the study. General exclusion criteria were contraindications for undergoing MRI, a history of (other) neurological or psychiatric disorders (apart from mild depression in the patient group, see below), less than full proficiency in speaking, reading, and writing German, and active medications known to affect the central nervous system or cognitive abilities except for acetylcholinesterase (AchE) inhibitors (11 subjects), memantine (1 subject + AchE inhibitor) or antidepressants (5 subjects, 4 + AchE-Inhibitor) in the patient group. Three subjects were excluded because of active corticoid-containing medications. Mild depression, a well-known comorbidity of AD, was not an exclusion criterion and was assessed using different scales: Geriatric Depression Scale (GDS) in 13 subjects (Yesavage et al., 1983), Rasch-based Depression Screening (DESC) I and II in 1 subject (Forkmann et al., 2009), and HAM-D in the remaining subjects. According to the test-specific cut-off values, 3 subjects in the AD group reported mild depressive symptoms (16 points on the HAM-D, 6 points and 8 points on the GDS).

Structural images (FLAIR and T1 sequences) were visually inspected by an interdisciplinary team of neurologists and neuroscientists experienced in dementia care and research. Subjects with extensive white matter lesions (Fazekas Score > 2 (Fazekas et al., 1987), 6 subjects) or relevant structural abnormalities (3 subjects: 1 large arachnoid cyst, 1 large post-ischemic lesion, 1 severe hydrocephalus) were excluded from further analyses.

A total of 66 subjects (29 healthy seniors, 37 patients with AD) were eligible for further analysis. To account for a significant age difference and uneven sample sizes, patients with AD were matched to HS for age using propensity score matching *via* an R-based custom dialog for SPSS 25 (Thoemmes, 2012), resulting in a final sample of 58 subjects (29 HS, 29 patients with AD).

**Figure 1:**
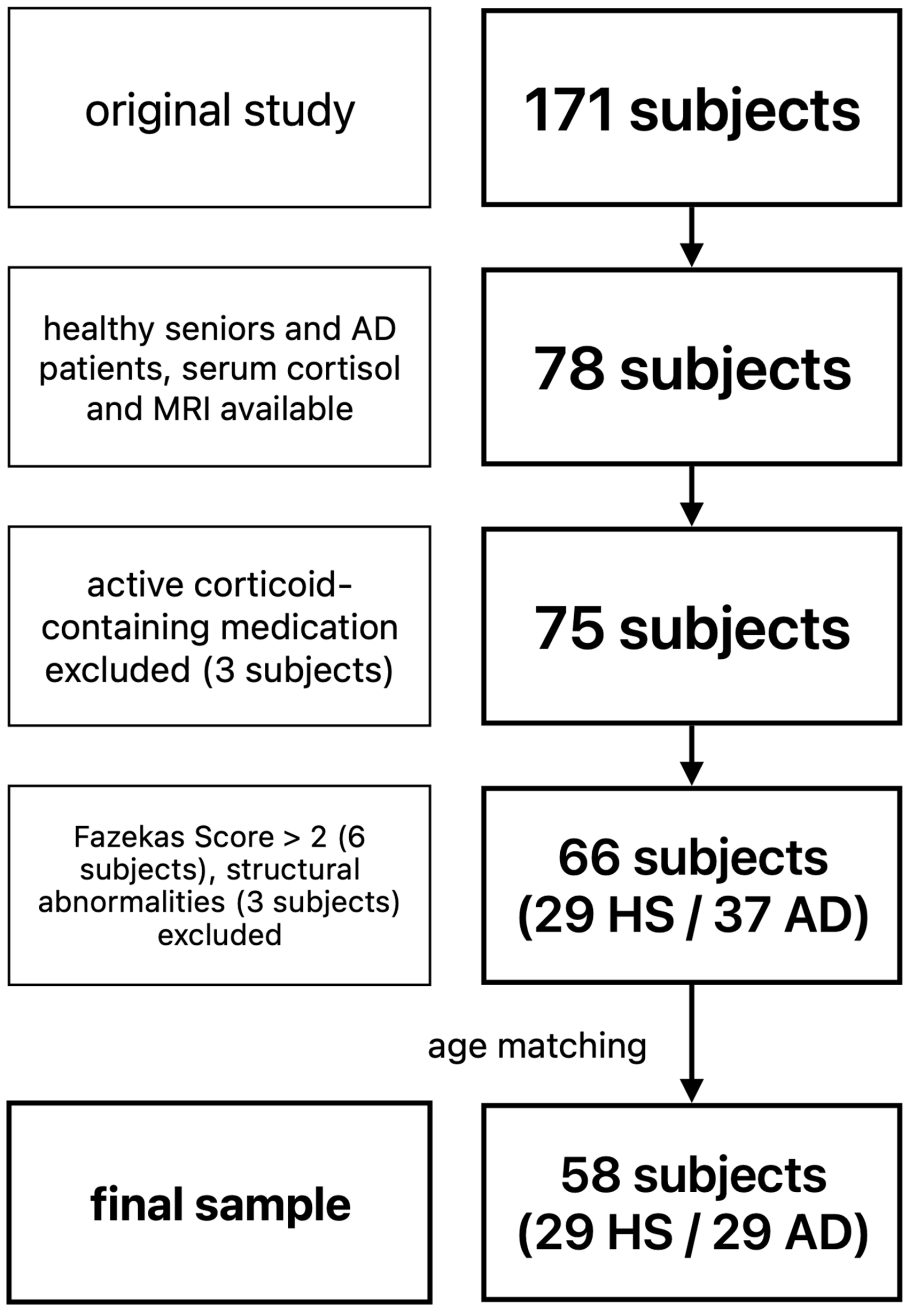
Flowchart of subject selection. HS, healthy seniors; AD, Alzheimer’s patients.

For detailed demographics see Table 1, and for a flowchart of subject selection Figure 1.

**Table 1:** Demographics.

	HS (*n* = 29)	AD (*n* = 29)	
Age (years)	63.17 (6.5)	66.07 (5.65)	*p* = 0.09
Sex (f/m)	9/20	14/15	*p* = 0.18
Education (years)	15.17 (4.27)	14.17 (3.47)	*p* = 0.332
Serum cortisol level (μg/L)	130.00 (43.36)	185.97 (61.48)	*p* < 0.001*
BMI (kg/m^2^)	25.46 (3.84)	23.17 (2.60)	*p* = 0.011*
Subjects with mild depression	0	3	*p* = 0.075
Verbal memory recall score (z-score)	0.85 (0.58)	−0.85 (0.4)	*p* < 0.001*
MMSE (/30)	29.52 (0.95)	24.10 (3.1)	*p* < 0.001*
Left Hippocampus (% TIV)	0.2730 (0.0278)	0.2032 (0.0295)	*p* < 0.001*
Right Hippocampus (% TIV)	0.2622 (0.0262)	0.2039 (0.03)	*p* < 0.001*
ApoE (% of at least one ε4 allele)	17.2	65.5	*p* < 0.001*
Handedness (% right)	89.7	96.6	*p* = 0.611

Mean and standard deviation, if applicable. HS, healthy seniors; AD, Alzheimer’s disease; BMI, Body-mass-index; MMSE, Mini-mental state examination score; TIV, Total intracranial volume; ApoE, Apolipoprotein E. * denotes significant results.

### 2.2. Standard protocol, approval, and consent

Every participant and, if applicable, a knowledgeable informant gave their written informed consent. Participants received compensation for participating, except for patients whose participation in the study was part of their diagnostic workup. In that case, we bore all costs related to the study including traveling costs and food.

All study procedures took place at the outpatient memory clinic, during an inpatient stay at the Department of Neurology, University Hospital Cologne or at the Jülich Research Centre. Except for one subject, in which neuropsychological evaluation was performed last, all subjects were evaluated in following order: neuropsychological evaluation, blood sampling, MR imaging. All MR scans were performed at the Jülich Research Centre.

Patients underwent a clinical examination by a trained neurologist at the University Hospital Cologne. A comprehensive neuropsychological examination partly used in this study was either performed during a visit at our outpatient memory clinic or during the inpatient diagnostic workup (the neuropsychological data used in this study was assessed not longer than 3 months apart from MR imaging). The inpatient workup consisted of a 2-day overnight stay at the Department of Neurology and included a lumbar puncture, a comprehensive neuropsychological evaluation, and blood sampling. Patients were transported to the Jülich Research Centre for the MR scan, at the end of their inpatient stay.

Healthy seniors were fully evaluated at the Jülich Research Centre (exceptionally neuropsychological evaluation was performed at the University Hospital Cologne due to subject preference).

Blood was drawn in the morning not later than noon on the same day as MR imaging took place in most of the cases (62.1% of subjects). In the remaining cases blood draw took place 1–19 days prior to MR imaging (mean = 4.86, SD 4.84).

Neuropsychological evaluation was performed at various times during normal working hours on the same day as MR scanning in 44.8% of subjects and 1–85 days prior in the remaining cases (mean 12.91, SD = 20.96). Neuropsychological evaluation and sampling of serum cortisol were performed on the same day in 79.3% of cases and 1–85 days apart in the remaining cases (mean = 25.67, SD = 30.2).

MR imaging took place from late morning to early afternoon.

The study was part of a more extensive study, approved by the local ethics committee (ref. no. 12–073), and carried out following the declaration of Helsinki.

### 2.3. Neuropsychology and behavioral analyses

All subjects underwent a comprehensive neuropsychological assessment previously described in detail (Dillen et al., 2016, 2017). We focused our analysis on the Verbal Learning and Memory Test (VLMT), the German version of the Rey Auditory Verbal Learning Test. More specifically, we narrowed our analyses to the recall after interference (run 6, R6) and the delayed recall (run 7, R7), as impaired memory recall is one of the initial and most prominent symptoms of AD (Tierney et al., 1996; Artero et al., 2003; Perri et al., 2007). In one AD subject, neuropsychological testing had to be aborted before the assessment of R7 due to exhaustion. The subject was subsequently assigned 0 points for the delayed recall (in line with the expected performance). Another AD subject was missing the values for R6 due to technical issues while recording the values. Little’s MCAR Test (Little, 1988) was performed to check if the data were missing completely at random and was not significant (*p* = 0.72). Completion *via* expectation–maximization was performed in the SPSS missing values module, and the estimated value was consistent with expectations based on other test results.

To increase variability and avoid ceiling and floor effects, a composite “verbal memory recall score” was created by averaging of the z-transformed values of R6 and R7.

All analyses were conducted using SPSS 25 (IBM Corp. Released 2017. IBM SPSS Statistics for Macintosh, Version 25.0. Armonk, NY: IBM Corp.). Data were checked for normality using the Shapiro–Wilk test. Accordingly, education, serum cortisol levels and body mass index (BMI) were compared by two-sample t-tests. Group differences in age, verbal memory performance, and MMSE scores were assessed using Mann–Whitney U tests. Chi-square tests (Fisher’s Exact tests when necessary) compared Apolipoprotein E (ApoE) status, handedness, sex, and depression. Results can be found in Table 1.

### 2.4. Cortisol

Morning serum cortisol levels were assessed *via* whole blood sampling (Sarstedt S-Monovette®, Serum Gel with Clotting Activator). Samples were refrigerated at the Jülich Research Centre before analysis in the central laboratory of the University Hospital Cologne, usually on the same day, if not analyzed directly on site in patients whose blood draw was part of their inpatient care at the University Hospital. Samples were analyzed using a commercially available competitive electrochemiluminescence immunoassay (Elecsys® Cortisol II, Roche Diagnostics). The Elecsys® Cortisol II assay makes use of a competition test principle using a monoclonal antibody explicitly directed against cortisol. Endogenous cortisol, liberated from binding proteins with danazol, competes with exogenous cortisol derivatives, labeled with ruthenium complex, for the binding sites on the biotinylated antibody. The measuring range as per the manufacturer’s information is 0.54–633.5 μg/L. All values for healthy seniors in our study fell within the normal range for morning values issued by the manufacturer (5th–95th percentile: 60.1–183.53 μg/L). The monthly inter-assay variation coefficients as assessed by the laboratory as part of their routine quality control measures were between 1.42–4.99% during the time of the study.

### 2.5. Structural imaging

#### 2.5.1. Data acquisition

Structural imaging was performed on a Siemens 3 T MAGNETOM Trio (Siemens, Erlangen, Germany). High-resolution T1-weighted structural images were acquired using a magnetization-prepared rapid gradient-echo sequence with the following parameters: Repetition time = 2,250 ms, echo time = 3.03 ms, flip angle = 9°, field of view = 256 × 256 mm^2^, matrix = 256 × 256, voxel resolution = 1 mm isotropic, 176 or 192 sagittal slices, no gap, slice order interleaved. A vacuum cushion was used to reduce head motion.

#### 2.5.2. Data preprocessing

Preprocessing of structural images was performed using the Computational Anatomy Toolbox (CAT12, Version 12.1, http://dbm.neuro.uni-jena.de/cat/), an extension of the Statistical Parametric Mapping software (SPM12, https://www.fil.ion.ucl.ac.uk/spm/software/spm12). CAT12 was used with default settings, described in detail in the CAT12 manual.^1^ In short, individual steps included bias correction, skull stripping, segmentation of brain tissue into gray matter (GM), white matter and CSF, spatial normalization of gray matter maps to the Montreal Neurological Institute (MNI) space using the Diffeomorphic Anatomical Registration Through Lie Algebra (DARTEL) (Ashburner, 2007), resampling to an isotropic resolution of 1.5 mm, and modulation of normalized gray matter images by their Jacobian determinants to preserve tissue volume. Finally, normalized gray matter maps were spatially smoothed using an isotropic gaussian kernel of 6 mm full width at half-maximum.

#### 2.5.3. Data analysis

Our analysis consisted of two parts: Analysis of hippocampal volumes in predefined atlas ROIs and whole-brain voxel-wise analysis.

The relationship between variables of interest (serum cortisol level, gray matter volume, and memory performance) and covariates (age, education, sex, presence of depression, and BMI) was evaluated beforehand, and covariates were chosen accordingly. BMI (Weiner et al., 1987; Fraser et al., 1999; Stalder et al., 2012) and depression (Keller et al., 2016; Høifødt et al., 2019) have previously been reported to relate to cortisol levels. However, in our sample, neither BMI nor the presence of mild depression (relevant only in the AD group) showed any significant relationship with serum cortisol levels and were thus disregarded in all further analyses.

To avoid any assumptions about the distribution of normality in the correlation analysis, we used Pearson’s correlation analysis with bootstrapping (1,000 samples), and bias-corrected accelerated 95% confidence intervals (BC_a_ CI) are reported throughout. Significance was assumed if BC_a_ CI did not include 0.

##### 2.5.3.1. Analysis of hippocampal volumes

CAT12 offers the possibility to estimate raw tissue volumes (in mm^3^) for different volume-based atlas maps implemented into the toolbox in native subject space before any spatial normalization. We chose the Automated Anatomical Labeling (AAL) atlas for all ROI-based analysis (Tzourio-Mazoyer et al., 2002). Left and right hippocampal volumes were extracted and corrected for total intracranial volume (TIV, as output by CAT12 Toolbox) using a simple ratio method (hippocampal volume/TIV*100). As a result, volumes were expressed as a percentage of TIV (%TIV). Left and right hippocampal volumes were correlated separately with cortisol levels across the whole sample and individual groups.

##### 2.5.3.2. Voxel-wise whole-brain analysis

All voxel-wise analyses were performed in SPM12 and conducted across the whole sample and both groups individually. Significant results are reported at an FWE-corrected cluster threshold of *p* < 0.05 (cluster forming threshold *p* < 0.001 uncorrected).

A multiple regression model was used to evaluate the relationship between serum cortisol levels and gray matter volume. Age, education, and TIV were included as covariates of no interest based on their significant relationships with serum cortisol levels (age and education) and gray matter volume (age). The creators of the CAT12 Toolbox generally recommend the inclusion of TIV as a covariate. Gray matter volume in mm^3^ was extracted from significant clusters for further analysis using a publicly available MATLAB script^2^ and corrected for TIV, using a simple ratio method (GM volume/TIV*100), subsequently expressing cluster values as a percentage of TIV (%TIV). The anatomical locations of significant clusters were determined *via* the SPM Anatomy Toolbox Version 2.2b (Eickhoff et al., 2005).

The relationship between cortisol levels and gray matter volume, as well as the correlation of gray matter volume and memory performance in the whole sample and in the individual groups, was further evaluated using correlation and partial correlation analysis in SPSS.

## 3. Results

### 3.1. Sample characteristics: cortisol, memory performance, hippocampus volume

**Figure 2:**
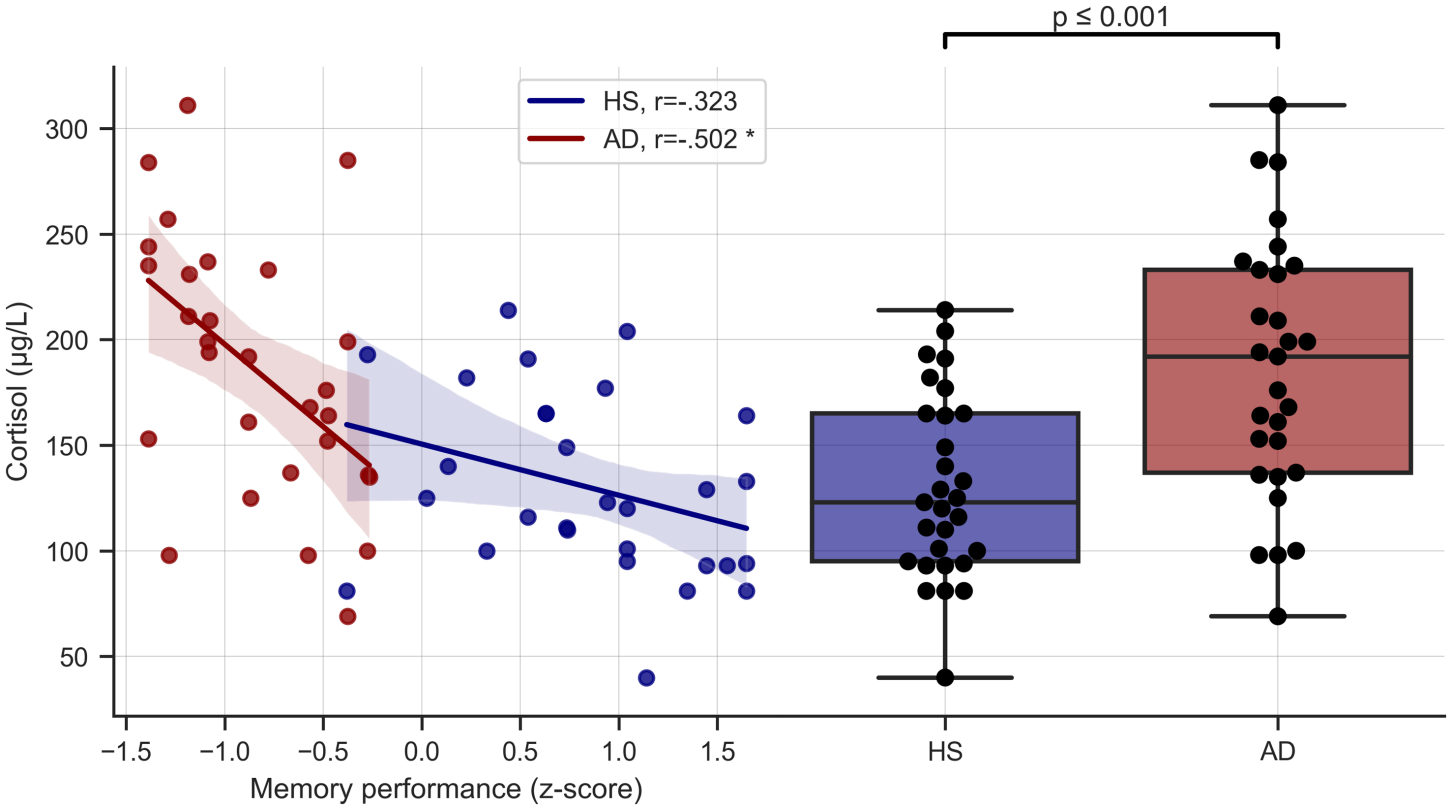
Cortisol levels and memory performance. Left side depicts unadjusted relationship of serum cortisol levels and memory performance, right side shows serum cortisol levels in the two groups. HS, healthy seniors; AD, Alzheimer’s patients, * = significant correlation, corrected for age and education.

Serum cortisol levels (μg/L) were significantly higher in the AD group (*M* = 185.97, *SD* = 61.48) than the HS group (*M* = 130, *SD* = 43.36), *t*(56) = 4.01, *p* < 0.001 (Figure 2). Group differences remained highly significant even when controlling for age, sex, BMI, education (though groups did not differ significantly in these covariates) and ApoE status [ANCOVA, *F*(1, 51) = 10.199, *p* = 0.002].

Across the whole sample, serum cortisol levels were correlated with age (*r* = 0.348, *p* = 0.007, *BCa 95% CI* [0.143, 0.524]) and education (*r* = −0.319, *p* = 0.015, *BCa 95% CI* [−0.493, −0.120]) but not with sex, in line with previous findings from subjects over 50 years of age (Roelfsema et al., 2017).

Verbal memory performance was significantly lower in patients with AD than HS (*p* < 0.001). Verbal memory performance was also significantly correlated with age (*r* = −0.407, *p* = 0.002, *BCa 95% CI* [−0.599, −0.152]), so that age and education were used as covariates of interest in subsequent analyses involving cortisol levels or memory performance.

As expected, right and left hippocampal volumes were significantly lower in patients with AD compared to HS [*t*(56) = −7.88 (right) / -9.27 (left), *p* < 0.001 respectively] and highly correlated with age (right: *r* = −0.448, *p* < 0.001, *BCa 95% CI* [−0.64, −0.216]; left: *r* = −0.439, *p* = 0.001, *BCa 95% CI* [−0.623, −0.196]).

### 3.2. Cortisol and memory performance

Verbal memory performance was negatively correlated with cortisol levels across the whole sample (*r* = −0.578, *p* < 0.001, *BC_a_ 95% CI* [−0.720, −0.409]) and in the AD group considered separately (*r* = −0.502, *p* = 0.006, *BC_a_ 95% CI* [−0.780, −0.165]), even when controlling for age and education (*r* = −0.479, *p* = 0.013, *BC_a_ 95% CI* [−0.764, −0.137]). Results remained unchanged when additionally controlling for ApoE carrier type. In the HS group there was a negative, albeit non-significant, trend (*r* = −0.323, *p* = 0.087, *BC_a_ 95% CI* [−0.616, 0.054]) that did not survive correction for age and education (*r* = −0.145, *p* = 0.471, *BC_a_ 95% CI* [−0.486, 0.193]) See Figure 2.

Interestingly, in the AD group, cortisol levels and memory performance were significantly correlated independent of hippocampal volumes (partial correlation, corrected for age, education, and left and right hippocampal volumes, *r* = −0.45, *p* = 0.024, *BC_a_ 95% CI* [−0.775, −0.041]), suggesting detrimental associations of higher cortisol with memory function independent or not wholly dependent on hippocampal involvement.

### 3.3. Analysis of hippocampal volumes

Across the whole sample right and left hippocampal volumes were negatively correlated with cortisol levels (right: *r* = −0.462, *p* < 0.001, *BC_a_ 95% CI* [−0.632, −0.264]; left: *r* = −0.573, *p* < 0.001, *BC_a_ 95% CI* [−0.723, −0.394]), even after correction for age and education (right; *r* = −0.359, *p* = 0.007, *BCa 95% CI* [−0.55, −0.184]; left: *r* = −0.513, *p* < 0.001, *BC_a_ 95% CI* [−0.679, −0.349]).

A similar relationship could be observed in HS, but with only the left hippocampus significantly associated with cortisol levels (*r* = −0.444, *p* = 0.016, *BC_a_ 95% CI* [−0.695, −0.158]) and the right side showing a non-significant trend (*r* = −0.328, *p* = 0.082, *BC_a_ 95% CI* [−0.628, 0.043]). After adding age and education as covariates, only the left hippocampal volume remained negatively related to cortisol levels (*r* = −0.398, *p* = 0.04, *BC_a_ 95% CI* [−0.597, −0.204]; right: *r* = 0.204, *p* = 0.307, *BC_a_ 95% CI* [−0.495, 0.069]). Results remained significant also after correcting for sex, handedness and ApoE genotype.

**Figure 3:**
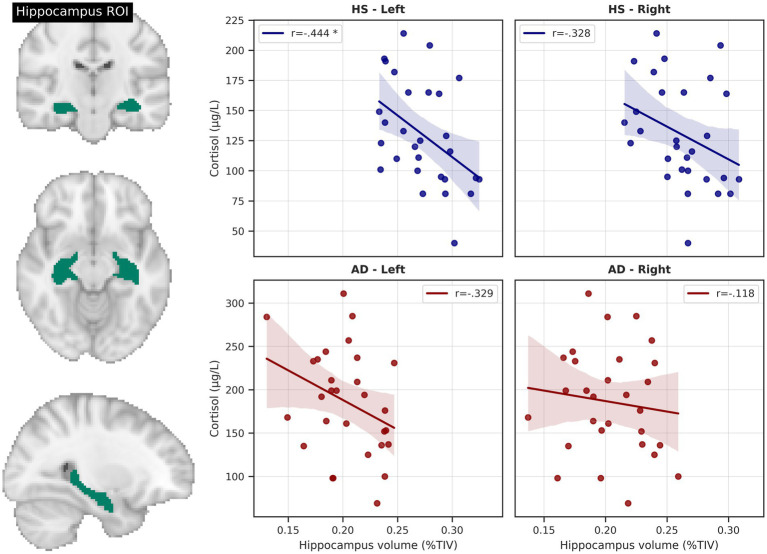
Hippocampus volume and cortisol levels. Scatterplots depict total intracranial volume (TIV)-corrected hippocampus volumes from AAL atlas region of interest (ROI) and unadjusted relationships with serum cortisol level. * = significant correlation, corrected for age and education. HS, healthy seniors, AD, Alzheimer’s patients.

In the AD group, the relationship between cortisol level and left hippocampal volume only showed a negative, albeit non-significant trend (*r* = −0.329, *p* = 0.082, *BC_a_ 95% CI* [−0.612, 0.008]). Conversely, there was no significant relationship with the right hippocampal volume (*r* = −0.118, *p* = 0.541, *BC_a_ 95% CI* [−0.465, 0.236]) See Figure 3.

In HS, left but not right hippocampal volume was positively correlated with memory performance (*r* = 0.458, *p* = 0.016, *BCa 95% CI* [0.112, 0.730], adjusted for age and education). No significant relationship could be detected in the AD group.

### 3.4. Mediation analysis – indirect effects of cortisol on memory performance in HS

**Figure 4:**
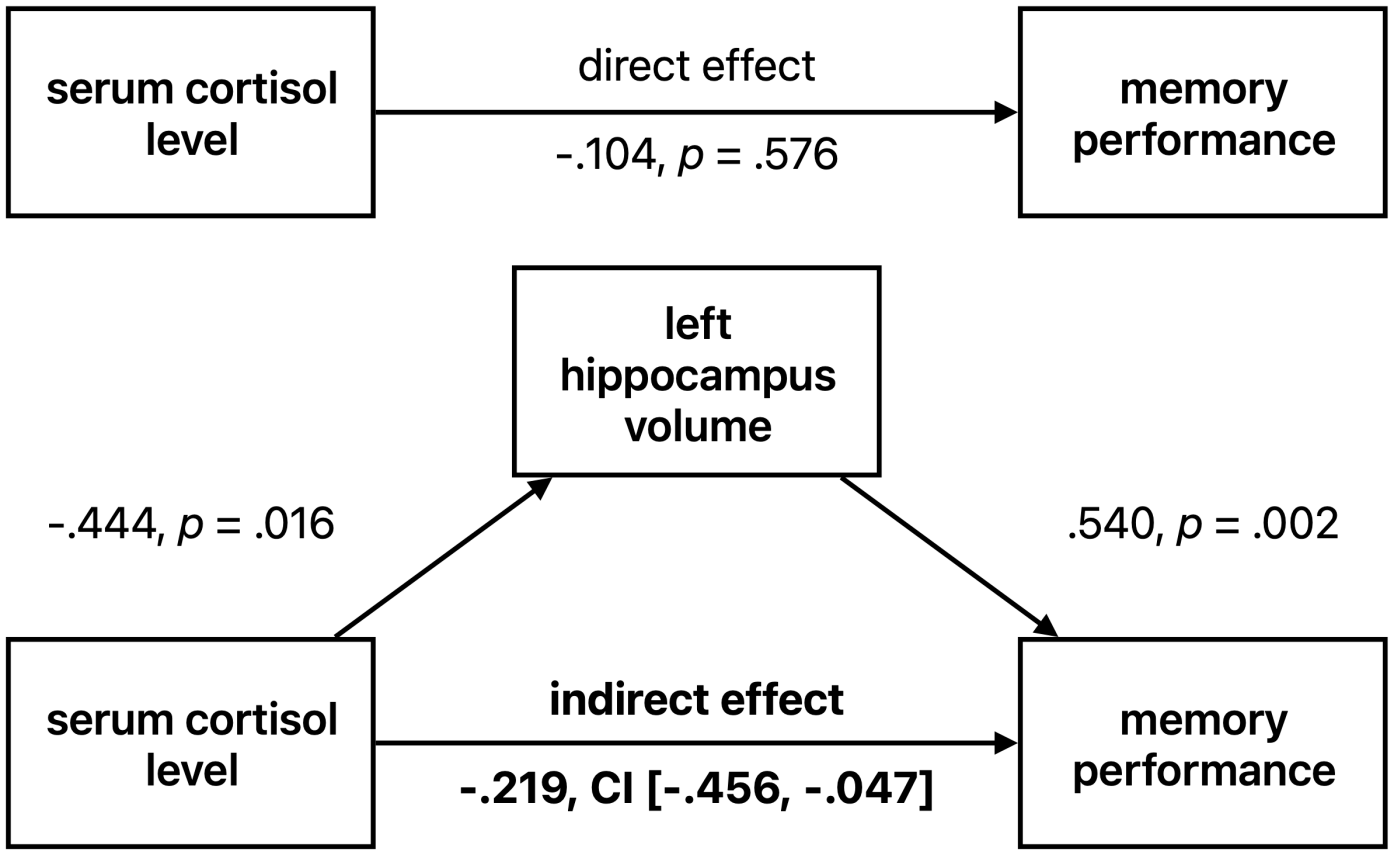
Left hippocampal volume mediating effect of cortisol on memory performance in healthy seniors. Standardized coefficients are reported. CI = 95% confidence interval.

**Figure 5:**
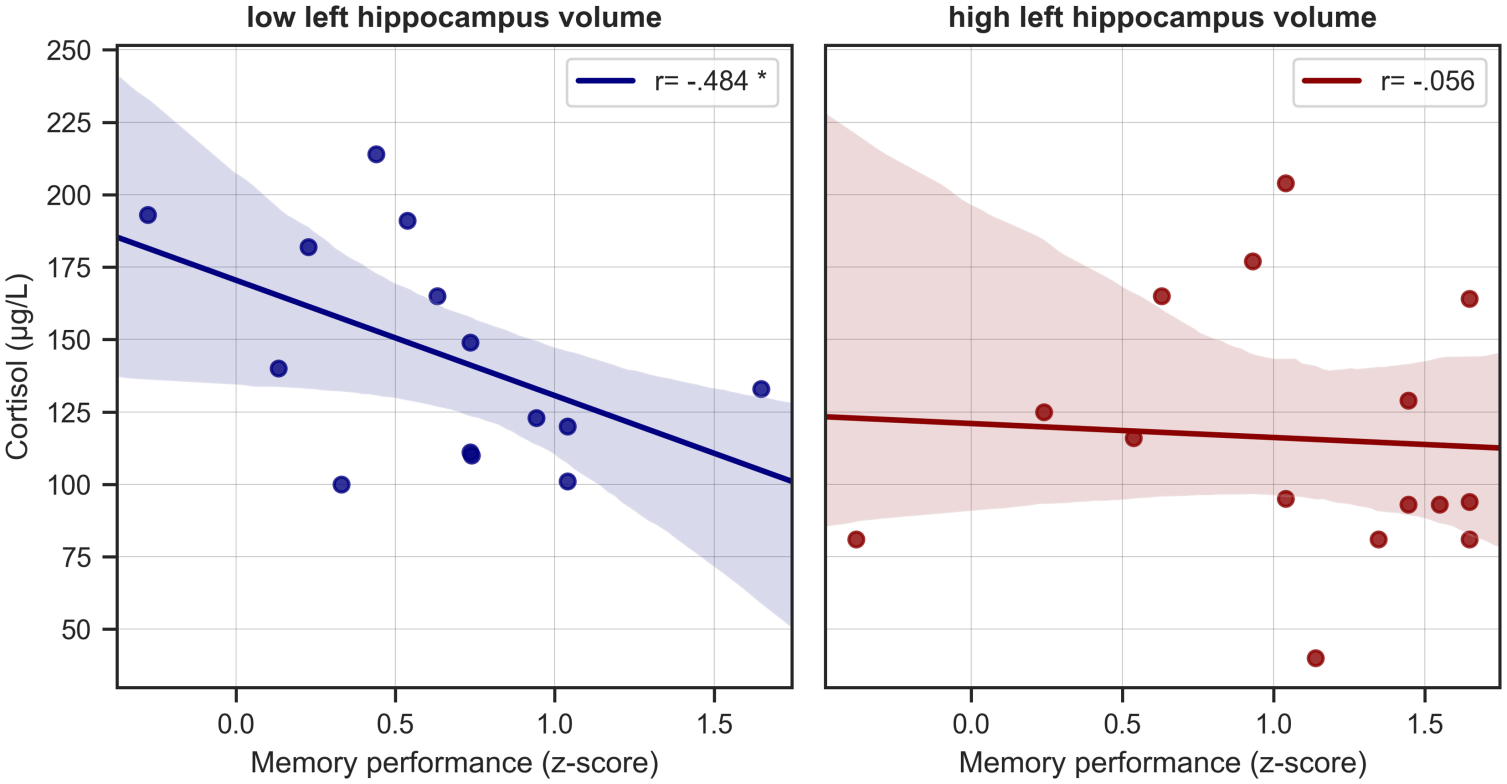
Cortisol levels and memory performance in healthy seniors split by hippocampal volume. Scatterplots depict unadjusted relationships between serum cortisol levels and verbal memory performance in healthy seniors split at the median into subjects with “low” and “high” hippocampus volume. * = significant correlation, corrected for age.

As both cortisol level and memory performance were significantly associated with left hippocampal volume in HS and given a negative trend (though not statistically significant) for the relationship between serum cortisol and memory performance, we tested whether the left hippocampal volume was possibly mediating the effect of serum cortisol on memory performance (Figure 4). We tested the significance of this indirect effect using the PROCESS macro for SPSS version 3.2 (Hayes, 2013) with model 4 (simple mediation). The bootstrapped standardized indirect effect (5,000 samples) was −0.219, *95% CI* [−0.469, −0.048]. Thus, the indirect serum cortisol effect on verbal memory performance *via* the left hippocampus volume was statistically significant. To corroborate this analysis, we performed a median split of left hippocampus volume (“low volume,” < 0.2707%/TIV, n = 14, “high volume,” > 0.2707%/TIV, n = 15). Subjects with lower hippocampal volume were significantly older than those with higher volume (on average by 5.88 years, two-sample t-test *p* = 0.012), but otherwise did not significantly differ in variables of interest (cortisol level, BMI, sex, memory performance). When analyzing the low and high volume groups separately, we found a significant negative relationship between cortisol level and memory performance in the low hippocampus volume group (corrected for age, *r* = −0.484, *p* = 0.094, *BCa 95% CI* [−0.817, −0.116]), but not in the high volume subjects (*r* = −0.056, *p* = 0.848, *BCa 95% CI* [−0.411, 0.435]) See Figure 5.

This further strengthens our results from the mediation analysis, that the negative association of cortisol levels with memory performance in healthy individuals is dependent on hippocampus volume.

### 3.5. Whole brain voxel-wise analysis

**Figure 6:**
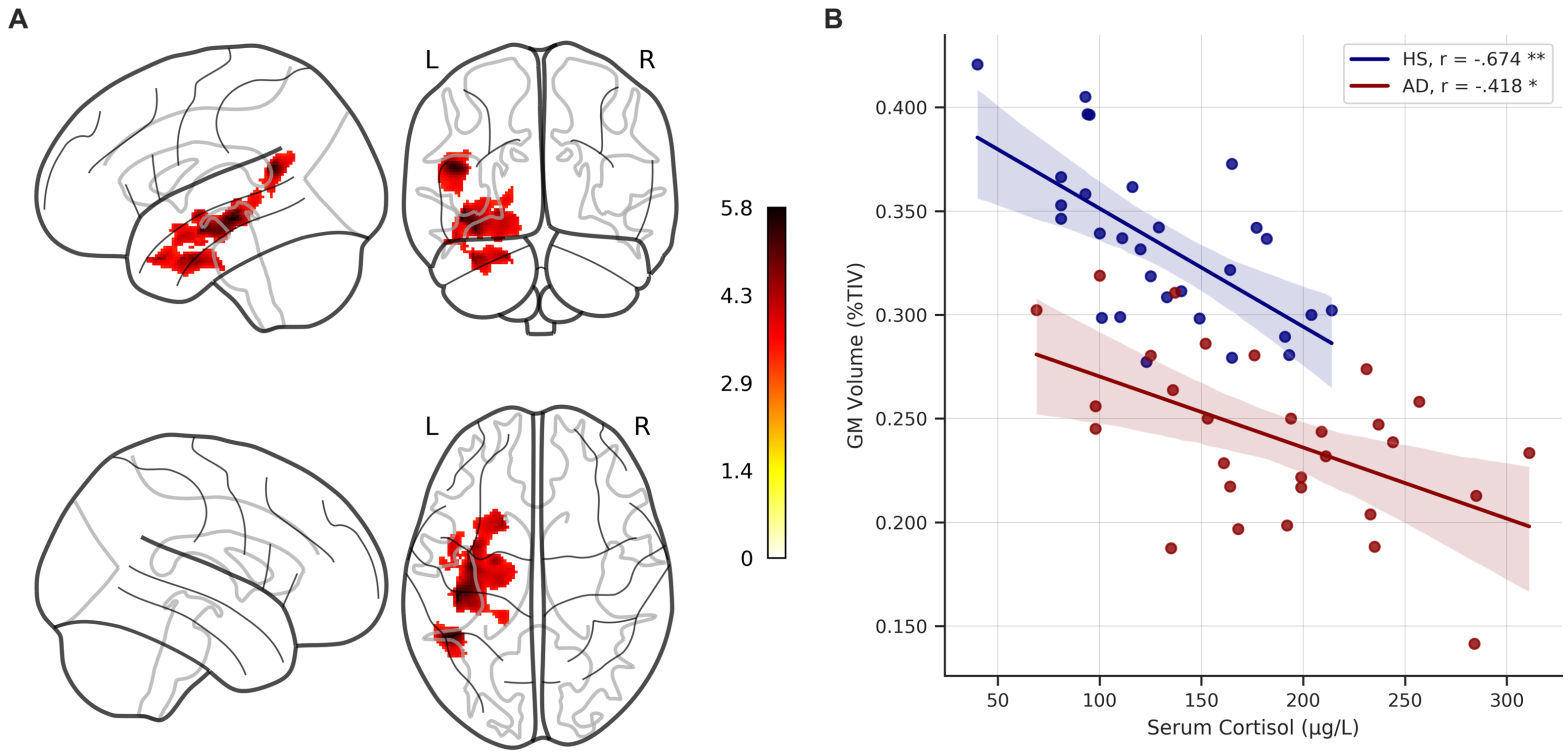
Voxel-wise correlation of serum cortisol levels and gray matter volume. **(A)** Glass brain showing clusters of significant negative correlation of serum cortisol levels and gray matter (GM) volume across the whole sample, corrected for age, education, and total intracranial volume (TIV) at *p* < 0.05 FWE-corrected (cluster-forming threshold *p* < *0*.001). Colorbar represents *t*-values. **(B)** Scatterplot depicts unadjusted relationship between serum cortisol levels and GM volume (adjusted for TIV and expressed as %TIV) in individual groups. * = significant correlation at *p* < 0.05, ** = significant correlation at *p* < 0.001, corrected for age and education, respectively. HS, healthy seniors; AD, Alzheimer’s patients; FWE, family-wise error.

Across the whole sample, serum cortisol levels were negatively correlated with gray matter volume in the hippocampus, the fusiform gyrus, the temporal pole, and the angular gyrus, and the middle temporal gyrus on the left hemisphere (cluster-forming threshold *p* < 0.001, *pFWE* < 0.05 at cluster-level, corrected for TIV, age and education, Figure 6, Table 2). No significant positive correlations between serum cortisol levels and gray matter volume were detected. Under the thresholds mentioned above, no significant voxel-wise results were found in the individual groups. Results remained largely unchanged when also correcting for sex, handedness and ApoE genotype.

**Table 2:** Significant clusters in voxel-wise analysis.

MNI Coordinates (x, y, z)	Cluster size (voxels)	Localization (all on left hemisphere)	peak T-value	*p*FWE (cluster-level)
−46, −48, 18	633	Angular gyrus, middle temporal gyrus	5.45	0.002
−42, −26, −10	2,248	Hippocampus	5.76	<0.001
−36, −4, −32	927	Fusiform gyrus, temporal pole	4.89	<0.001

MNI, Montreal Neurological Institute; FWE, family-wise error.

The extracted and TIV adjusted GM volumes were correlated with serum cortisol levels in the individual groups, correcting for age and education. Significant negative correlations were detected in both groups (HS: *r* = −0.674, *p* < 0.001, *BCa 95% CI* [−0.838, −0.43]; AD: *r* = −0.418, *p* = 0.03, *BCa 95% CI* [−0.677, −0.035]). The correlation strengths (corrected for age and education) did not differ significantly between groups (Fisher r-to-z transformation, *z* = −1.344, *p* = 0.089).

## 4. Discussion

Our present study investigated the relationships of serum cortisol levels, memory performance, hippocampal volume, and whole-brain voxel-wise gray matter volume in a sample of healthy seniors and patients along the AD spectrum.

Our main findings were that 1) cortisol levels were significantly elevated in patients with AD as compared to HS, 2) higher cortisol levels were associated with worse memory performance in patients with AD, 3) higher cortisol levels were associated with smaller left hippocampal volume in HS and there was an indirect negative effect of higher cortisol levels on memory performance mediated by left hippocampal volume – i.e., worse memory performance in subjects with higher cortisol levels and smaller hippocampi, and 4) higher cortisol levels were associated with lower gray matter volume not only in the hippocampus but also in temporal and parietal areas in the left hemisphere in both groups.

### 4.1. Serum cortisol

Serum cortisol levels were significantly higher in patients with AD than HS, a finding in line with previous publications [for a recent meta-analysis and review see (Zheng et al., 2020)]. Along with the glucocorticoid cascade hypothesis, this could be explained by loss of inhibitory control of the hippocampus over the HPA-axis due to hippocampal damage and atrophy caused by AD pathology, a process accelerated over time by glucocorticoid toxicity on the hippocampus, i.e., a vicious circle (Sapolsky et al., 1986).

Due to the cross-sectional design of our study, our findings do not allow for the assumption of any causal relationship between elevated cortisol levels and disease, a common problem among comparable studies. Due to cognitive impairment and consecutively higher stress and anxiety levels, higher cortisol levels in the AD group may have been caused by study-related procedures, an effect previously reported for MRI scanning (Tessner et al., 2006) and neuropsychological testing (Sindi et al., 2013), and which may be especially pronounced in older subjects (Lupien et al., 2007). Many other aspects such as different sleeping patterns have been shown to influence cortisol levels (Petrowski et al., 2020) and may have been an issue not accounted for. Though this also holds true for healthy elderly controls, the effect might have been more substantial in the AD group. However, other recent studies demonstrated longitudinal associations between higher cortisol levels and the future onset of AD (Hinterberger et al., 2013; Ennis et al., 2017), thereby corroborating the notion of a disease-related process and not just a stress-induced epiphenomenon. Furthermore, our findings of negative associations between cortisol levels and brain structure (see the following paragraphs) in both healthy seniors and patients with AD strengthen this point and argue for an observation not solely ascribable to stress or anxiety, making the notion of a pure epiphenomenon even more unlikely.

### 4.2. Serum cortisol, hippocampal volume, and memory performance

Cortisol levels have frequently been found to be inversely correlated with memory performance in healthy older adults (Li et al., 2006; Kukolja et al., 2008; Geerlings et al., 2015; Ouanes et al., 2017; Echouffo-Tcheugui et al., 2018), individuals with mild cognitive impairment (Wolf et al., 2002; Popp et al., 2015), and patients with AD (Csernansky et al., 2006). Also, glucocorticoid administration has been shown to impair declarative memory in healthy adults (Newcomer et al., 1994, 1999). However, these effects seem to be dose-dependent with higher cortisol levels (in comparison to a control group or condition and thereby probably often “medium” levels) even supporting better memory performance under certain circumstances (Kukolja et al., 2008, 2011), presumably reflecting an inverse U-shaped relationship between cortisol levels and memory performance (Schilling et al., 2013). This effect could be due to a differential occupancy of two types of cortisol receptors in the brain (i.e., Type I mineralocorticoid receptors, MR, and Type II glucocorticoid receptors, GR) (Lupien et al., 2007).

While we could only detect a significant direct association between cortisol levels and memory performance in the AD group but not in the HS sample, higher cortisol levels seem to be linked to worse memory function in HS indirectly *via* hippocampal volume. Also, in those subjects with smaller left hippocampi considered separately, we observed significant associations between cortisol levels and memory performance. This finding is remarkable, as hippocampal atrophy is a significant hallmark of AD and smaller hippocampus volumes serve as a risk factor for AD (Walhovd et al., 2020). Therefore, apparently healthy subjects with smaller left hippocampal volume seem to exhibit similar associations between serum cortisol and memory function as patients with AD do. It could be argued that the deleterious effects of cortisol on hippocampal architecture and, subsequently, memory performance will lead these individuals towards AD, a process that, at the moment, is still asymptomatic, but could be the start of the “glucocorticoid cascade” according to the hypothesis (Sapolsky et al., 1986). However, due to the cross-sectional design of this study, we could not further elaborate on this issue. In addition, no biomarker data were available for healthy subjects. Therefore, it cannot be excluded that HS with smaller hippocampal volumes were already harboring Alzheimer’s pathology.

Of note, in the AD group, there was no significant correlation between serum cortisol and hippocampal volume in the ROI analysis, and the negative association between cortisol levels and memory performance was independent of hippocampal volumes, suggesting that the hippocampus is not necessarily the central structure mediating adverse effects of cortisol levels on memory performance in the disease state. As mentioned above, different states of receptor occupancy concomitant with different involvement of limbic and prefrontal areas may play an essential part here: Under the condition of saturated MR receptors, a more substantial involvement of GR receptors will be in place, not only affecting the hippocampus, but also prefrontal regions that contain only GR receptors (Lupien et al., 2007). This effect may have been more pronounced in AD than HS due to the overall higher cortisol levels in patients. Also, our voxel-wise analysis demonstrated negative associations between cortisol levels and gray matter volume in left-hemispheric regions extending beyond the hippocampus, impairment of which may have also contributed to cognitive dysfunction. Interestingly, none of these included prefrontal areas.

It is also possible that the association between glucocorticoids and hippocampal structure is only relevant in earlier stages of toxicity and damage (Kulstad et al., 2005; Green et al., 2006). However, this seems somewhat contradictory to our voxel-wise analysis, which (still) demonstrated negative associations between cortisol levels and hippocampal gray matter volume in patients with AD. The higher spatial specificity of voxel-wise analysis may have played a role here.

### 4.3. Serum cortisol and voxel-wise gray matter volume

Our findings from the voxel-wise analyses not only corroborate the results from the ROI-based analysis – a negative relationship between cortisol levels and hippocampal architecture – but further extend them by also demonstrating negative associations between cortisol levels and gray matter volume in structures extending beyond the mesial temporal lobe, i.e., temporal and parietal cortex.

While some studies have also found negative relationships between cortisol levels and gray matter volume in parietal (Toledo et al., 2013; Lebedeva et al., 2018; Wirth et al., 2019), (pre)frontal (Toledo et al., 2013; Echouffo-Tcheugui et al., 2018), and occipital areas as well as with total brain volume (Echouffo-Tcheugui et al., 2018), others could not demonstrate such effects beyond the hippocampus (Lawlor et al., 1994; Lupien et al., 1998; Huang et al., 2009). A limiting factor in the latter and other similar studies may have been the *a priori* selection of regions of interest precluding more widespread findings, while the former – including our own present study – used a whole-brain approach.

Two aspects of our results are especially noteworthy: Firstly, the negative associations between cortisol levels and gray matter volume were found in brain regions strongly implicated in AD, i.e., (medial) temporal and parietal cortex (Whitwell et al., 2012; Ferreira et al., 2020). While for the AD group it could be argued that these areas are already incapacitated and prone to further damage through glucocorticoid toxicity, this cannot be argued for HS, showing no signs of the disease. Secondly, the correlation strength did not differ significantly between groups, suggesting that detrimental effects of cortisol on gray matter volume in otherwise apparently healthy elderly adults are similar if not equal to the corresponding mechanisms in AD. Taken together, this argues for a relationship independent of the presence of symptomatic AD.

One can only speculate whether molecular aspects such as tau and amyloid depositions are a common denominator, e.g., if higher cortisol levels accelerate the accumulation of toxic proteins and thereby gray matter atrophy in symptomatic and healthy or asymptomatic subjects alike. Advanced imaging methods such as amyloid and tau PET (Villemagne et al., 2021) might be able to unravel the role of cortisol levels on the complicated interrelationship of age, protein deposition, and clinical phenotype and offer a promising field for further studies.

### 4.4. Lateralization of association between serum cortisol and measures of brain structure

Notably, in both our ROI-based and voxel-wise analysis, significant associations between serum cortisol, memory performance, and brain structure were detected on the left hemisphere only, suggesting a left-lateralized vulnerability of the implicated structures and mechanisms.

While the mediating effect of left hippocampal volume on the association between cortisol levels and memory performance could be explained by the verbal nature of the task and thereby the primary involvement of the left hemisphere, this does not explain the “task-free” association between cortisol levels and gray matter volume. Another possibility may have been a patient selection bias by only including patients with AD with attested verbal memory impairment and thereby, indirectly, predominantly left hemispheric alterations. However, the same lateralized associations could be detected in healthy seniors without memory impairment.

Left and right hemispheric lateralization in the context of stress and cortisol effects has been discussed before. However, these findings are far from clear [for a recent review, see (Ocklenburg et al., 2016)]. Considering hippocampal volume in particular, studies are reporting negative associations of cortisol levels with both left (Wirth et al., 2019), right (Rajagopalan et al., 2020), and bilateral hippocampi (Toledo et al., 2013; Nguyen et al., 2019; Rajagopalan et al., 2020). Furthermore, the role of lateralization remains uncertain, as not all studies report associations with individual hippocampal volumes, but instead used a combined bilateral approach, e.g., (Starkman et al., 1992; O’Brien et al., 1996; Lupien et al., 1998; Huang et al., 2009; Knoops et al., 2010). To gain deeper insights into the question of possibly lateralized effects, further studies with separate inclusion of the left and right hippocampi are warranted. However, it is noteworthy that the lateralization of negative associations between serum cortisol and brain structure in our study further strengthens the hypothesis that elevated cortisol levels are a disease-specific marker rather than an epiphenomenon caused by, e.g., study-related procedures. If we assume that AD causes anxiety, stress, and thereby elevated cortisol levels (which in turn would only represent the AD pathology underlying anxiety and stress), one would expect a more generalized and bilateral pattern of cortical associations, resembling the one typically found in AD compared to healthy subjects.

### 4.5. Strengths and limitations

One of the significant strengths of our study is the thorough diagnosis of AD based on biomarkers (CSF and PET). However, no biomarkers were available for HS. Therefore, it cannot be excluded that some subjects – though cognitively unimpaired – were harboring preclinical Alzheimer’s disease with incipient changes in cortisol levels and gray matter structure.

Only three other studies used voxel-wise analyses (Toledo et al., 2013; Echouffo-Tcheugui et al., 2018; Wirth et al., 2019) with our study being the only one combining ROI-based analyses, voxel-wise analyses, and assessment of memory performance.

Some of the more recent studies included larger samples of a few hundred to a few thousand subjects, analyzing data from large cohort studies such as ADNI (Toledo et al., 2012, 2013; Wirth et al., 2019) the Framingham Heart Study (Echouffo-Tcheugui et al., 2018), BLSA (Ennis et al., 2017) or AGES-Reykjavik (Geerlings et al., 2015). We were able to confirm findings from these studies in a smaller, yet independent sample. Sex distribution in the studies cited above was comparable to our own female study population of ~40%, ranging from ~40–60%. Compared to our study with a mean participant age of ~65 years the studies based on the ADNI and AGES dataset had a higher mean age of ~75 years, while in the Framingham Heart Study analysis (including only healthy participants, mean age 48.5 years) and the BLSA sample (~60 years) participants were younger.

However, we only evaluated cortisol levels at one time point in a cross-sectional manner. Therefore, no conclusion about the temporal sequence of events is possible (especially concerning higher cortisol levels in patients with AD in the setting of study-related procedures) and diurnal variations of cortisol secretion cannot be accounted for. Also, it would be helpful to assess different measures of cortisol (e.g., plasma, serum, urine, CSF, saliva, hair) to account for method-associated differences – in our case because the major portion of serum cortisol is bound to cortisol-binding globulin and albumin and only a fraction represents unbound free cortisol assumed to be biologically active (Slaunwhite et al., 1962; Coolens et al., 1987) – and to investigate acute versus chronic effects (e.g., hair cortisol is assumed to reflect chronic stress). Also, it would be useful to include other AD phenotypes (e.g., logopenic variant, posterior cortical atrophy) to study associations not only attributable to verbal memory impairment.

## 5. Conclusion

Our study offers further insight into the relationships between serum cortisol, brain structure, and memory function by not only demonstrating negative associations of higher cortisol levels with gray matter volume and verbal memory performance in patients with AD but also showing similar adverse effects in apparently healthy elderly subjects, especially in those with smaller hippocampal volume.

By possibly acting as a common denominator among associations between AD, memory, sleep, depression, and physical activity – all of which were been shown to have some association with glucocorticoids – the study of cortisol offers a promising field for future research and therapeutic opportunities in healthy aging and disease.

## Data availability statement

The raw data supporting the conclusions of this article will be made available by the authors, without undue reservation.

## Ethics statement

The studies involving human participants were reviewed and approved by the Ethics Commission of Cologne University’s Faculty of Medicine, Cologne, Germany. The patients/participants provided their written informed consent to participate in this study.

## Author contributions

OO, JK, and HJ designed the original study. JD, NR, and KD collected the data. JD and AO analyzed the data and JD wrote the manuscript. OO, JK, AO, HJ, GB, QB, NR, RF, HG, KD, and GF revised and approved the manuscript. All authors contributed to the article and approved the submitted version.

## Funding

HG was supported by the Cologne Clinician Scientist Program (CCSP)/Faculty of Medicine/University of Cologne, funded by the German Research Foundation (DFG, FI 773/15–1). GF receives royalties from the publication of the books “Funktionelle MRT in Psychiatrie und Neurologie,” “Neurologische Differentialdiagnose,” and “SOP Neurologie”; receives royalties from the publication of the neuropsychological tests KAS and Köpps; received honoraria for speaking engagements from Bayer, Desitin, DGN, Ergo DKV, Forum für medizinische Fortbildung FomF GmbH, GSK, Medica Academy Messe Düsseldorf, Medicbrain Healthcare, Novartis, Pfizer, and Sportärztebund NRW. This study received funding from the Brandau-Laibach-Foundation (JK and OO) and the Marga and Walter Boll-Foundation (JK, GF, and OO). The funders were not involved in the study design, collection, analysis, interpretation of data, the writing of this article or the decision to submit it for publication.

## Conflict of interest

GF serves as an editorial board member of Cortex, Neurological Research and Practice, NeuroImage: Clinical, Zeitschrift für Neuropsychologie, and DGNeurologie.

The remaining authors declare that the research was conducted in the absence of any commercial or financial relationships that could be construed as a potential conflict of interest.

## Publisher’s note

All claims expressed in this article are solely those of the authors and do not necessarily represent those of their affiliated organizations, or those of the publisher, the editors and the reviewers. Any product that may be evaluated in this article, or claim that may be made by its manufacturer, is not guaranteed or endorsed by the publisher.

## Footnotes

^1^http://www.neuro.uni-jena.de/cat12/CAT12-Manual.pdf

^2^http://www0.cs.ucl.ac.uk/staff/g.ridgway/vbm/get_totals.m
